# An Efficient Pipeline Wavefront Phase Recovery for the CAFADIS Camera for Extremely Large Telescopes

**DOI:** 10.3390/s100100001

**Published:** 2009-12-24

**Authors:** Eduardo Magdaleno, Manuel Rodríguez, José Manuel Rodríguez-Ramos

**Affiliations:** Departmento de Física Fundamental y Experimental, Electrónica y Sistemas, University of La Laguna, Avd. Francisco Sanchez s/n, 38203 La Laguna, Spain; E-Mails: mrvalido@ull.es (M.R.); jmramos@ull.es (J.M.R.-R.)

**Keywords:** plenoptic sensors, wavefront sensors, adaptive optics, real-time processing, FPGA

## Abstract

In this paper we show a fast, specialized hardware implementation of the wavefront phase recovery algorithm using the CAFADIS camera. The CAFADIS camera is a new plenoptic sensor patented by the Universidad de La Laguna (Canary Islands, Spain): international patent PCT/ES2007/000046 (WIPO publication number WO/2007/082975). It can simultaneously measure the wavefront phase and the distance to the light source in a real-time process. The pipeline algorithm is implemented using Field Programmable Gate Arrays (FPGA). These devices present architecture capable of handling the sensor output stream using a massively parallel approach and they are efficient enough to resolve several Adaptive Optics (AO) problems in Extremely Large Telescopes (ELTs) in terms of processing time requirements. The FPGA implementation of the wavefront phase recovery algorithm using the CAFADIS camera is based on the very fast computation of two dimensional fast Fourier Transforms (FFTs). Thus we have carried out a comparison between our very novel FPGA 2D-FFTa and other implementations.

## 1. Introduction

The resolution of ground-based astronomical observations is strongly affected by atmospheric turbulence above the observation site. In order to achieve resolution close to the diffraction limit of the telescopes, AO techniques have been developed to offset wavefront distortion as it passes through turbulent layers in the atmosphere.

AO includes several steps: detection of the phase gradients, wavefront phase recovery, information transmission to the actuators and their mechanical movement. The next generation of extremely large telescopes (from 50 to 100 meter diameters) will demand significant technological advances to maintain the segments of the telescopes aligned (phasing of segmented mirrors) and also to offset atmospheric aberrations. For this reason, faster wavefront phase reconstruction seems to be of utmost importance, and new wavefront sensor designs and technologies must be explored. The CAFADIS camera presents a robust optical design that can meet AO objectives even when the references are extensive objects (elongated LGS and solar observations). The CAFADIS camera is an intermediate sensor between the Shack-Hartmann and the pyramid sensor. It samples an image plane using a microlens array. The pupil phase gradients can be obtained from there, and after that, the phase recovery problem is the same as in the Shack-Hartman.

In this work, our main objective is to select a good and fast enough wavefront phase reconstruction algorithm, and then to implement it over the FPGA platform, paving the way for accomplishing the computational requirements of the ELT’s number of actuators within a 6 ms limit, which is atmospheric response time.

The modal estimation of the wavefront consists in using the slope measurements to fit the coefficients of an aperture function in a phase expansion of orthogonal functions. These functions are usually Zernike polynomials or complex exponentials, but there are other possibilities, depending on the pupil mask. Very fast algorithms can be implemented when using complex exponential polynomials because the FFT kernel is the same [1,2]. Zonal reconstruction consists in estimating a phase value directly instead of the coefficients of an expansion, and they require an iterative process. The modal algorithms produce more precise results than the zonal solution and –this is crucial- are suited to parallelization. Consequently, this is the preferred estimation to accomplish the phase reconstruction on technologically advanced platforms such as FPGAs or graphical processing units (GPUs) [3]. Once the algorithm based on the expansion over complex exponential polynomials has been selected, an efficient FFT implementation in FPGA is the core of an optimal phase reconstruction.

We will start by describing the modal Fourier wavefront phase reconstruction algorithm, and how the Fast Fourier Transform tallies with the FPGA architecture, analyzing the obtained efficiency and comparing it to implementations in other technologies and platforms. We design an initial 64 × 64 full pipeline phase recovery prototype using the synthesized 2D-FFT module. The system was satisfactorily circuit-tested using simulation data as phase gradients. Finally, we analyze the obtained efficiency and compare it to the modal wavefront using high-end CPU.

## 2. Background

**Figure 1.**
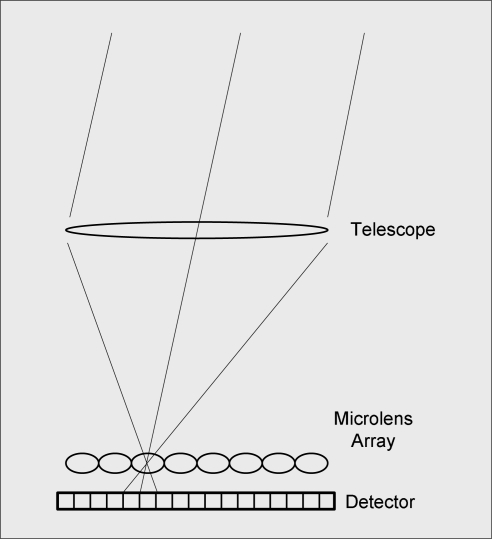
Outline of the Plenoptic camera used as wavefront sensor.

**Figure 2.**
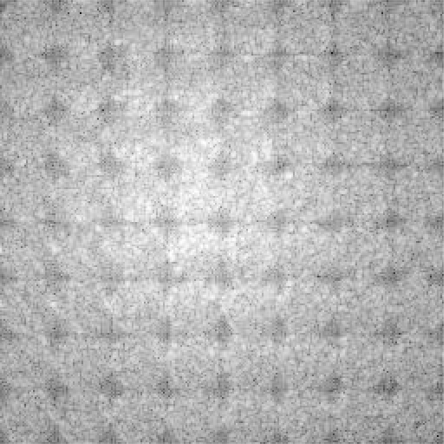
Section of the plenoptic frame showing the LGS on axis. The remaining, off-axis LGS present a similar aspect.

The CAFADIS plenoptic sensor samples the signal ψ*_telescope_*(*u,v*) (complex amplitude of the electromagnetic field) to obtain the wavefront phase map *ϕ* (*u,v*). A microlens array is placed at the focal point of the telescope (as in a pyramid sensor), sampling the image plane instead of the pupil plane (as in a Shack-Hartmann sensor). If the f-numbers of both telescope and microlens are the same, the detector will be filled up with images of the pupil of maximum size without overlapping. Wavefront phase gradients at telescope pupil plane are extracted from the plenoptic frame taken by the CAFADIS, and wavefront maps from different viewpoints are obtained. Hence, tomographic wavefront reconstruction could be accomplished from only one plenoptic frame (Figure 1) [4]. For example, Figure 2 shows a section of the plenoptic frame obtained for CAFADIS, containing data from five artificial laser guide stars (data simulation assuming a 10 m diameter telescope, and every subpupil sampled by 32 × 32 pixels [4]). With this information, the CAFADIS camera has the capability of refocusing at several distances and selecting the best focus as object distance.

The final phase resolution depends on the number of pixels sampling each microlens, but depth resolution also depends on the same quantity. This implies that, increasing the phase resolution, higher height resolution is obtained at the same time. In the extreme case, when using a pyramid sensor (2 × 2 microlens), the phase and height resolution are maximized. At the other extreme, when using a Shack-Hartmann wavefront sensor, the phase resolution depends on the number of subpupils, and height resolution is minimized (and even lost).

A compromise solution might be taken: a unique plenoptic sensor, comprised by 6 × 6 subpupils sampled by 84 × 84 pixels would be enough to get phases with 84 × 84 pixel resolution using only one 504 × 504 pixels detector. Or even, in order to avoid detector contamination due to the neighboring LGS images, the plenoptic image could be sampled by 12 × 12 subpupils. In this case, a 1,008 × 1,008 detector is needed [4].

The phase gradients at pupil plane are calculated from this plenoptic frame using the partial derivates of the wavefront aberration estimated in [5] and implemented in FPGA in [6]. From these gradient estimation, the wavefront phase *ϕ* (*u*,*v*) can be recovered using an expansion over complex exponential polynomials, allowing application of 2D-FFT:

(1) $$\phi (u,v)=\sum_{p,q=0}^{N-1}a_{\mathrm{pq}}\;Z_{\mathrm{pq}}\;(u,v)=\sum_{p,q=0}^{N-1}a_{\mathrm{pq}}\frac{1}{N}e^{\frac{2\pi i}{N}(\mathrm{pu}+\mathrm{qv})}=\mathrm{IFFT}(a_{\mathrm{pq}})$$

The gradient is then written:

(2) $$\vec{S}(u,\;v)=\vec{\nabla }\phi (u,\;v)=\frac{\partial \phi }{\partial u}\vec{i}+\frac{\partial \phi }{\partial v}\vec{j}=\sum_{p,q}a_{\mathrm{pq}}\vec{\nabla }Z_{\mathrm{pq}}$$

Making a least squares adjustment over the *F* function:

(3) $$F=\sum_{u,\;v=1}^{N}{\left[\vec{S}(u,\;v)-\sum_{p,q}a_{\mathrm{pq}}(\frac{{\partial Z}_{\mathrm{pq}}}{\partial u}\vec{i}+\frac{{\partial Z}_{\mathrm{pq}}}{\partial v}\vec{j})\right]}^{2}$$

where *S⃗* are experimental data, the coefficients *a_pq_* of the complex exponential expansion in a modal Fourier wavefront phase reconstructor (spatial filter) can be written as:

(4) $$a_{\mathrm{pq}}=\frac{i\mathrm{pFFT}\left\{S^{x}(u,\;v)\right\}+\mathrm{iqFFT}\left\{S^{y}(u,\;v)\right\}}{p^{2}+q^{2}}$$

The phase can then be recovered from the gradient data by reverse transformation of the coefficients:

(5) $$\phi (u,\;v)={\mathrm{FFT}}^{-1}[a_{\mathrm{pq}}]$$

A filter composed of three two-dimensional Fourier transforms therefore must be calculated to recover the phase. In order to accelerate the process, an exhaustive study of the crucial FFT algorithm was carried out which allowed the FFT to be specifically adapted to the modal wavefront recovery pipeline and the FPGA architecture.

## 3. Control System

**Figure 3.**
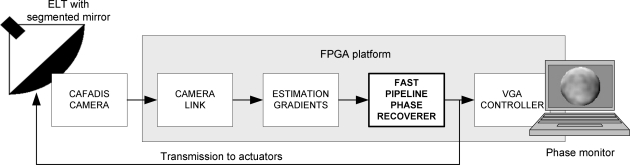
Modules of the control system.

The global control system to be developed is shown in Figure 3. The functional architecture has three sub-modules. At the front of the system the camera link module receives data from CAFADIS in a serial mode. The following stages perform the digital data processing using FPGA resources. The estimation of the phase gradients is a very simple computation which can be conducted using correlation or Clare-Lane algorithms [5,6]. The main computing power is carried out by the wavefront phase recovery. Thus, efficient implementation is crucial in order to carry out the loop within the atmospheric time limit for the new ELTs and our work has centered its efforts on this module. Finally, the estimated recovered phase can be monitored via a VGA controller.

## 4. Algorithm to Hardware

We will focus on the FPGA implementation from Equations (4) and (5) to improve processing time. These equations can be implemented using different architectures. We could choose a sequential architecture with a single 2D-FFT module where data values use this module three times in order to calculate the phase. This architecture represents an example of an implementation using minimal resources of the FPGA. However, we are looking for fast implementation of the equations in order to stay within the 6 ms limit of the atmospheric turbulence. Given these considerations, we chose a parallel, totally pipeline architecture to implement the algorithm. Although the resources of the device increase considerably, we can maintain time restrictions by using extremely high-performance signal processing capability through parallelism. We therefore synthesize three 2D-FFTs instead of one 2D-FFT.

**Figure 4.**
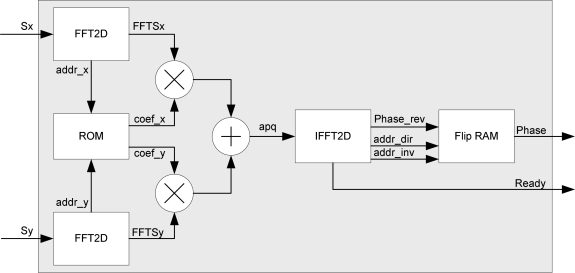
Architecture of the synthesized phase recoverer.

The block diagram of the designed recoverer is depicted in Figure 4 where *Sx* and *Sy* represent the image displacement into each subpupil. The two-dimensional transforms of *Sx* and *Sy* have to be multiplied by $\frac{\mathrm{ip}}{p^{2}+q^{2}}$ and $\frac{\mathrm{iq}}{p^{2}+q^{2}}$ respectively according to Equation 4. These two matrices are identical if we exchange rows and columns. We can therefore store a single ROM. The results of the adders (*a_pq_* coefficients) are rounded appropriately to obtain 16 bits data precision according with the data input width of the inversed two-dimensional transform that is executed at the next stage.

An analysis of the equations and a parallel architecture of its implementation are taken into account. We then break down the design into the following steps or stages:

1. Compute two real forward 2D FFT that compute FFT (*Sx*) and FFT (*Sy*)
2. Compute the complex coefficients
3. Carry out a complex inverse 2D FFT on *a_pq_*
4. Flip data results

### 4.1. Architecture of FFT Module

Generally, each butterfly implies one complex multiplier and two complex adders. In particular, multipliers consume much silicon area of FPGA because they are implemented with adder trees. Various implementation proposals have been made to save area by removing these multipliers [7–10].

However, in order to implement an efficient multiplier, the last Virtex-4 FPGA devices incorporate specific arithmetic modules, called DSP48. Each DSP48 slice has a two-input multiplier followed by multiplexers and a three-input adder/subtractor. With these circuits, the FPGA only needs four clock cycles to calculate the complex multiplication with up to 550 MHz in XC4VSX35 Virtex-4 [11,12].

**Figure 5.**
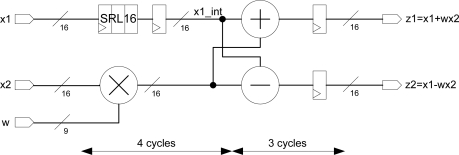
Pipeline radix-2 butterfly in FPGA.

The complete pipeline radix-2 butterfly can be implemented with this specialized multiplier. It is necessary to use a FPGA Look-Up Table (LUT) (configured as SRL16 shift register) to preserve the synchronism. The butterfly implemented is depicted in Figure 5 and it needs only seven clock cycles to carry out the computation.

A pipeline radix-2 FFT can be implemented using one butterfly at each stage. The twiddle coefficients used in each stage are stored in twiddle LUT ROMs in the FPGA. The logic resources and the clock cycles of the FFT module is reduced in our implementation using specific butterfly modules at the first and second stages. The first stage utilizes the feature of the twiddle factors related to the first stages of the pipeline:

(6) $$W_{N}^{N/2}=1$$

So, the first stage can be implemented in a very simple way with an adder/subtractor. In the second stage, the next twiddle factors are:

(7) $$W_{N}^{N/4}=j$$

This twiddle suggests a similar splitting structure in the second pipeline stage as in the first one; however, the imaginary unit imposes a special consideration: two additional multiplexers change real and imaginary data, and the pipeline adder/subtractor works according to Equation 8:

(8) (a+bj)j=aj−b=−b+aj

**Figure 6.**
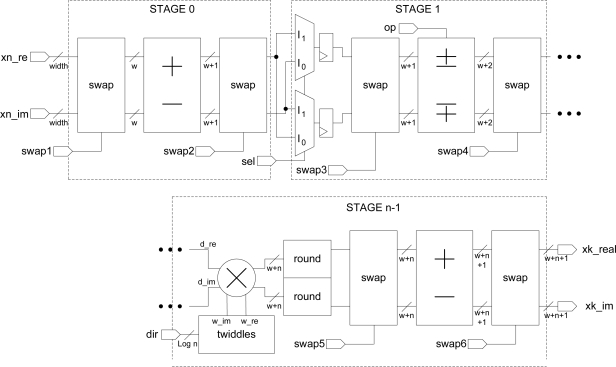
Architectural block diagram of a pipeline radix-2 FFT.

Taking into account these features, the 1D-FFT architecture implementation is depicted in Figure 6. The swap-blocks arrange the data flow and preserve the pipeline feature. It consists of two multiplexers and two shift registers. These shift registers are implemented using look-up tables (LUT) in shift register mode (SRL16) for synchronization.

The system performs the calculation of the FFT with no scaling. The unscaled full-precision method was used to avoid error propagations. This option avoids overflow situations because output data have more bits than input data. Data precision at the output is:

(9) $$\mathrm{output}\;\mathrm{width}=\mathrm{input}\;\mathrm{width}+{\text{log}}_{2}\;\;\mathrm{points}+1$$

The number of bits on the output of the multipliers is much larger than the input and must be reduced to a manageable width with the use of one-cycle symmetric rounding stages (Figure 6). The periodic signals of the swap units, *op* signal, and the address generation for the twiddle memories are obtained through a counter module that acts as control unit.

### 4.2. Temporal Analysis for the Radix-2 FFT Module and Superior Radix

Taking into account the clock cycles of each block in Figure 6, the latency of the FFT module can be written as:

(10) $$\mathrm{latency}=2\left(\frac{N}{2}+1\right)+2+1+2\left(\frac{N}{4}+1\right)+2+\cdots +\sum_{n=2}^{({\text{log}}_{2}N)-1}\left[7+2\left(\frac{N}{2^{n+1}}+1\right)\right]$$

where the first two stages are considered separately, and *N* and *n* are the number of points of the transform and the number of stages of the module respectively. Adding the geometrical series and grouping, finally:

(11) $$\mathrm{latency}=2N+9{\text{log}}_{2}\;N-11,\;\;\;N=8,\;16,\;32,\;\ldots$$

When the number of points of the FFT is a power of 4, it is computationally more efficient to use a radix 4 algorithm instead of radix 2. The reasoning is the same as in radix 2 but subdividing iteratively a sequence of *N* data into four subsequences, and so on. The radix-4 FFT algorithm consists of log_4_*N* stages, each one containing *N*/4 butterflies. As the first weight is $W_{N}^{0}=1$, each butterfly involves three complex multiplications and 12 complex sums. Performing the sum in two steps, according to [13], it is possible to reduce the number of sums (12 to 8). Therefore, the number of complex sums to be performed is the same (*N*log_2_*N*) as the algorithm in base 2, but the multipliers are reduced by 25% (of (*N*/2) log_2_*N* to (3*N*/8) log_2_*N*). Consequently, the number of circuits for DSP48 use is reduced proportionally.

When the number of points is a power of 4, the pipeline radix-4 FFT module has half the arithmetic stages, but the swap modules need twice the amount of clock cycles to arrange the data. Then, the latency is expressed as:

(12) $$\mathrm{latency}(\mathrm{radix}4)=2N+9{\text{log}}_{4}\;N-13,\;\;\;N=16,\;64,\;256,\;1024,\;\ldots$$

This time estimation has been conducted for other radix, as shown in the following equations:

(13) $$\begin{array}{l} \mathrm{latency}(\mathrm{radix}\;8)=2N+9{\text{log}}_{8}\;N-21,\;\;\;N=64,\;512,\;\ldots \\ \mathrm{latency}(\mathrm{radix}\;16)=2N+9{\text{log}}_{16}\;N-37,\;\;\;N=2^{8},\;2^{12},\;\ldots \end{array}$$

Generalizing:

(14) $$\mathrm{latency}(\mathrm{radix}\;i)=2N+9{\text{log}}_{i}\;N-5-2i,\;\;\;\;\begin{array}{c} N=i^{n+1},\;n=1,\;2,\;3,\;\ldots \\ i=4,\;8,\;16,\;\ldots \end{array}$$

**Figure 7.**
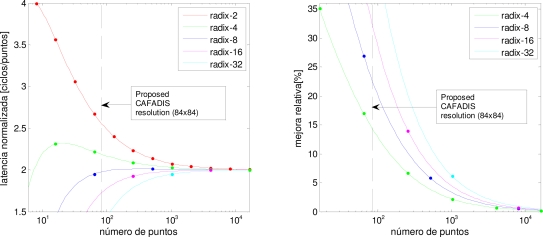
(a) Normalized latency. (b) Relative improvement regarding radix-2 algorithm.

Figure 7 shows the clock cycles of each algorithm and the proposed CAFADIS resolution with a vertical line. All implementations are close to 2 when the number of points grows (Figure 7a). The improvement in terms of computing speed of the algorithm using other radix is relevant when the number of samples is small. For example, the improvement factor for a 1,024-point FFT is less than 7% using a radix-32 algorithm and less than 3% using a radix-4 (Figure 7b). However, in our astronomical case the proposed size is relatively small and the improvement using superior radix is relevant. Examining Figure 7b, we can observe that the improvement factor is about 20% using radix-8 and 30% using radix-16. Thus, we are considering the implementation of these algorithms in the future.

### 4.3. Comparison with Other Implementations

**Figure 8.**
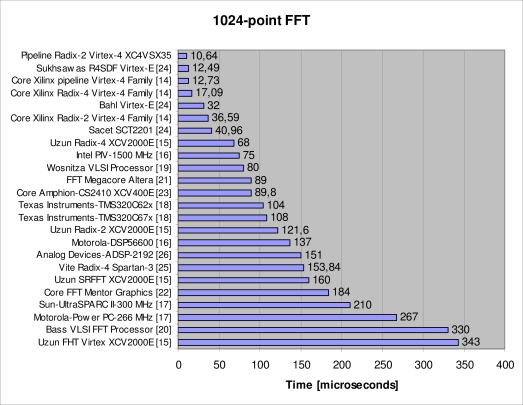
Execution times in microseconds for various algorithms of 1024-points FFT using different technologies.

Several radix-2 FFT were satisfactorily synthesized in a XC4VSX35 Virtex-4 FPGA. A comparison has been carried out between our design and other implementations. The combined use of the FPGA technology and the developed architecture achieves an improved performance if compared to other alternatives. This is shown in Figure 8 where our implementation executes a 1,024-point FFT operation in 10.64 μs.

### 4.4. 64 × 64 2D-FFT Implementation

For a first prototype of the phase recoverer, we have selected a plenoptic sensor with 64 × 64 pixels sampling each microlens. The fundamental operation in order to calculate the corresponding 64 × 64 2D-FFT is equivalent to applying a 1D-FFT on the rows of the matrix and then applying a 1D-FFT on the columns of the result. Traditionally, the parallel and pipeline algorithm is then implemented in the following four steps:

1. Compute the 1D-FFT for each row
2. Transpose the matrix
3. Compute the 1D-FFT for each column
4. Transpose the matrix

**Figure 9.**
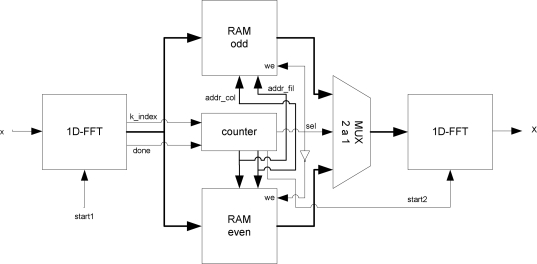
Block diagram of the implemented 2D-FFT.

Figure 9 depicts the diagram of the implemented transform module. The operation of the developed system takes place when image data is received in serial form by rows. These data are introduced in a block that carries out a one dimensional FFT. As this module obtains the transformed data, the information is stored in two double-port memories (real and imaginary data). To complete the two-dimensional FFT, the stored data is introduced in a second 1D-FFT in column format. The 2DFFT is then obtained from the output of this block.

Continuous data processing using a single dual-port memory (real and imaginary) is not possible. Therefore, the new transformed data must wait for the old data to be introduced in the second FFT block, otherwise data are overwritten. As a result, the pipeline property of the FFT architecture cannot be used. This problem can be avoided by using two memories instead of one, where memories are continuously commuting between write and read modes. When the odd memory is reading and introducing data values in the second FFT module, the even memory is writing data which arrives from the first FFT. So, data flow is continuous during all of the calculations in the two-dimensional transform. The memory modes are always alternating and the function is selected by the counter. The same signal is used to commute the multiplexer that selects the data entering the column transform unit.

It is worth mentioning that the transposition step defined above (Step 2) is implemented simultaneously with the transfer of column data vector to the memory with no delay penalty. In this way, the counter acts as an address generation unit. The last transposition step (Step 4) is not implemented in order to save resources and obtain a fast global system. So, the last transposition step is taken into account only at the end of the algorithm described in Equations (4) and (5) and shown in Figure 4 (Flip-RAM module).

Row 1D-FFT block and column 1D-FFT block are not identical due to the unscaled data precision. So, a 64 × 64 2D-FFT for the phase recoverer must meet certain requirements. If the precision of data input is 8 bits, the output data of 1D-FFT of the rows has to be 15 bits according Equation (9). 1D-FFT of the columns accepts a 15 bits data format and 22 bits at the output.

Several FFTs were implemented over a XC4VSX35 Virtex-4 device and numerical results were satisfactorily compared with MatLab simulations. As we show in Equations (4) and (5), three 2D-FFTs are needed to implement a pipeline wavefront phase reconstructor. It can only recover the phase with a sensor up to 64 × 64 subpupils using 8 bits precision and up to 32 × 32 with 16 bits precision. This size is sufficient at this time for the prototype; however, if we want to implement a greater recoverer, we should select an FPGA with more resources.

Taking into account the latency of the FFT (Equation 15) and the pipeline operation of the memory modules, the latency of the 2D-FFT module can be written as:

(15) $$\mathrm{latency}=N^{2}+4N+18\;{\text{log}}_{2}\;N-22,\;\;\;\;\;N=8,\;16,\;32,\;\ldots$$

Table 1 shows a performance comparison of existing 2D-FFTs implementations using FPGA and others technologies for matrix sizes 64 × 64 and 128 × 128. Rodríguez-Ramos *et al.* [3] implemented 2D-FFT on a CPU AMD XP 3500+, 2211 GHz, with 512 KB L2 cache and on a GPU nVidia GeForce 7800 GTX graphical engine with 256 MB RAM. Uzun *et al.* [15] implemented several algorithms on a Virtex-2000E FPGA chip where the fastest design is depicted in the table. Evidently, our design shows improvements when compared to [3] and [15] in terms of frame rate performance.

## 5. Results, Analysis and Comparative Study

**Figure 10.**
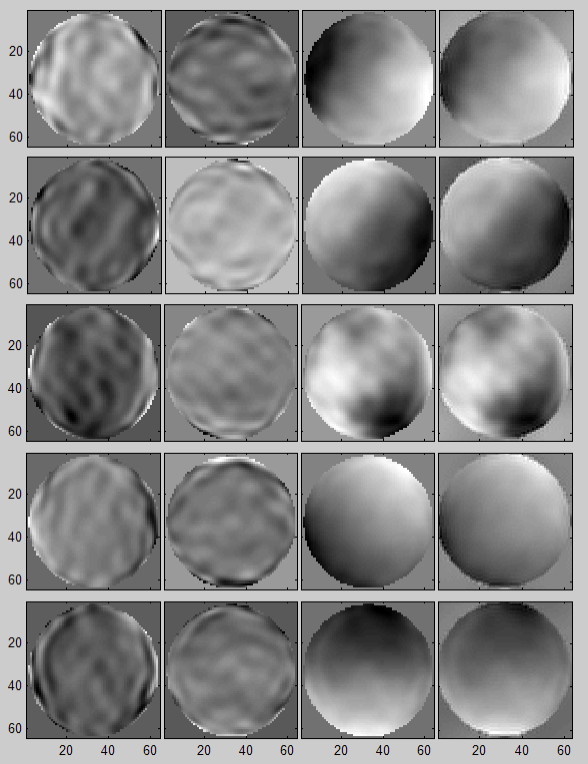
Phase gradients and original and recovered phase for a CAFADIS camera with 64 × 64 subpupils.

The design of a 64 × 64 phase recoverer was programmed using the VHDL hardware description language [17] and XST was used to synthesize these modules [18] into a XC4VSX35 Virtex-4 FPGA. The complete system was successfully circuit-tested using ChipScope Pro software (using phase gradients obtained in simulations) that directly inserts a logic analyzer and bus analyzer into the design, allowing any internal signal to be viewed. Signals are captured at operating system speed and brought out through the programming interface. Captured signals can then be analyzed with the PC that acts as a logic analyzer. The numeric results were also successfully compared with those obtained in Matlab. Figure 10 shows the results of several wavefront reconstructions using a 64 × 64 subpupil recoverer. Each row shows the phase gradients (Sx, Sy) given to the module. Below are the incoming phases at the CAFADIS camera and, finally, the phase obtained with the algorithm implementation in the Virtex-4 where the error is less than 3.5%.

Table 2 shows the total time broken down into the stages of the total system (depicted in Figure 4). 12,980 clock cycles are necessary for phase recovery, going from data reception to the activation of the *ready* signal. This value is the latency time for the phase recoverer. At a 100 MHz frequency clock, the system needs less than 130 μs to recover the phase. Table 3 shows Virtex-4 resource utilization and the maximum operating frequency (pre-synthesis).

The implemented architecture is pipeline. This architecture allows phase data to be obtained for each 4,096 clock cycles (this number coincides with the number of points of the transforms, that is, the number of subpupils, 64 × 64, of the CAFADIS camera). Using the 100 MHz clock, the prototype provides new phase data each 40.96 μs.

These results can be compared with other works. Rodriguez-Ramos *et al.* implemented a 64 × 64 phase recoverer using GPU [3]. In this technology, the wavefront reconstruction needs 3.531 ms. The FPGA implementation results almost 30 times faster. Baik *et al.* [28] implemented a wave reconstruction with a 14 × 14 Shack-Hartmann array, an IBM PC and a Matrox Meteor-2 MC image processing board. The wavefront correction speed of the total system was 0.2 s. Although the system includes the gradient estimation, it can be seen that the execution times are slower than in the proposed implementation. Seifer *et al.* [29] used a sensor with 16 × 12 subpupils and a Pentium M, 1.5 GHz. The wavefront reconstruction in this case was 50 ms using Zernike polynomials to adjust to the coefficients of the aperture function. Again, our implementation using FPGA technology is comparatively faster.

## 6. Conclusions

A 64 × 64 wavefront recoverer prototype was synthesized with a Xilinx XC4VSX35 Virtex-4 as sole computational resource. This FPGA is provided in a ML402 Xtreme DSP evaluation platform. Our prototype was designed using ISE Foundation 8.2 and ModelSim 6.0 simulator. The system has been successfully validated in the FPGA chip using simulated data.

A two-dimensional FFT is implemented as nuclei algorithm of the recoverer: processing times are really short. The system can process data in much lower times than the atmospheric response. This feature allows more phases to be introduced in the adaptive optical process. Then, the viability of the FPGAs for AO in the ELTs is assured.

Future work is expected to be focused on the optimization of the 2D-FFT using others algorithms (radix-8, radix-16) and the implementation of a larger recoverer into Virtex-5 and Virtex-6 devices for the necessary 84x84 recoverer using CAFADIS camera. The prototypes could be four times faster than with Virtex-4 FPGA devices. Moreover, the system should be tested in a telescope expected soon.

## Figures and Tables

**Table 1.** 2D-FFT performance comparison with other designs.

**2D-FFT**	**Rodríguez-Ramos *et al.* [3]**		**FPGA Uzun *et al.* [15]**	**FPGA Proposed**
	**CPU**	**GPU**		
**64 × 64**	114.5 μs	1,580 μs	-	44.4 μs
**128 × 128**	811.0 μs	1,680 μs	2,380 μs	170.8 μs

**Table 2.** Execution time (latency) for the different stages of the phase recoverer.

**Module**	**Cycles**	**Duration (@ 100MHz)**
2D-FFT (Sx and Sy)	4,438	44.38 μs
Multipliers	4	0.04 μs
Adder	1	0.01 μs
2D-IFFT	4,438	44.38 μs
Flip-RAM	4,096	40.96 μs
Rounding (3)	3	0.03 μs
**Total**	12,980	129.8 μs

**Table 3.** Virtex-4 resources.

**Slices**	**Slices FFs**	**4-LUT**	**IOB**	**BRAM**	**DSP48**	**Fmax**
13993 (91%)	13201 (62%)	19478 (63%)	39 (8%)	74 (38%)	53 (27%)	189.519 MHz
